# Titanium Biohybrid Middle Ear Prostheses: A Preliminary *In Vitro* Study

**DOI:** 10.3390/jfb14120561

**Published:** 2023-11-29

**Authors:** Mario Mosconi, Elena Carlotto, Laura Caliogna, Micaela Berni, Giulia Gastaldi, Michele Conti, Alice Maria Brancato, Valentina Bina, Domenico Minervini, Stefano Malpede, Anna Chiara Stellato, Francesco Lazzerini, Luca Bruschini, Marco Benazzo, Pietro Canzi

**Affiliations:** 1 Department of Clinical, Surgical, Diagnostic and Pediatric Sciences, University of Pavia, 27100 Pavia, Italy; 2 Orthopedics and Traumatology Clinic, IRCCS Policlinico San Matteo Foundation, 27100 Pavia, Italy; 3 Department of Otorhinolaryngology, University of Pavia, IRCCS Policlinico San Matteo Foundation, 27100 Pavia, Italy; 4 Department of Molecular Medicine, University of Pavia, 27100 Pavia, Italy; 5 Centre for Health Technologies, University of Pavia, 27100 Pavia, Italy; 6 Department of Civil Engineering and Architecture (DICAr), University of Pavia, Via Ferrata 3, 27100 Pavia, Italy; 7 Otolaryngology, ENT Audiology and Phoniatrics Unit, University Hospital of Pisa, 56124 Pisa, Italy

**Keywords:** middle ear, ossicular replacement prosthesis, titanium, bone transplantation, tissue engineering, hearing loss

## Abstract

Ossiculoplasty is a surgical operation performed to restore auditory transmission through the reconstruction of the ossicular chain using prosthetics. Tissue bioengineering has assumed a pivotal role in implementing alternatives to conventional ossicular middle ear replacement prostheses, to overcome extrusion while preserving acoustic properties. This *in vitro* study aims to explore, for the first time in current literature, the feasibility of a biohybrid middle ear prosthesis, composed of titanium surrounded by a bone extracellular matrix as bio-coating. We have hereby studied the adhesion and proliferation of human adipose-derived mesenchymal stem cells (hASC) on titanium scaffolds *in vitro*. Moreover, we identified the osteogenic differentiation of hASC using an immunofluorescence assay to analyze osteoblasts’ gene expression profiles (Alp, Runx2, Col1a1, Osx, and Bglap), and we counted the presence of collagen as a marker of hASC’s ability to secrete an extracellular matrix. We utilized scanning electron microscopy to evaluate the presence of an extracellular matrix on the scaffolds. Our preliminary data demonstrated the titanium’s ability to support human adipose-derived mesenchymal stem cell colonization, proliferation, and osteoblastic differentiation, in order to obtain a biohybrid device. Our experience seems encouraging; thus, we advocate for further *in vivo* research to corroborate our results regarding bone transplantation.

## 1. Introduction

The modern approach to middle ear (ME) surgery, which combines both disease removal and functional reconstruction, dates back to the 1950s [1]. Ossiculoplasty (OPL) is an established surgical procedure intended to restore appropriate sound-wave transmission from the tympanic membrane to the oval window via a reconstructed ossicular chain [2]. It often represents the rehabilitative stage after eradication of various ME pathologies, including chronic otitis media, adhesive otitis media, atelectasis, tympanosclerosis, tumors, traumatic injuries, and congenital diseases, which may damage the chain and lead to conductive hearing loss [3]. The functional results are variably associated with surgical techniques, host factors, and prostheses features [4]. The ideal ossiculoplasty material should not only be safe and steadily inbuilt into surrounding tissues, but it should also provide hearing restoration. Over the past decades, several materials have been evaluated for these purposes with varying degrees of success. Historically, the first reconstructions were performed using autograft ossicles [5]. Still today, autologous incus refashioned to provide customized interposition remains one of the reconstruction techniques of choice due to its rare extrusion and low cost. Unfortunately, the availability and quality of patients’ incus are often limited in diseased ears [2]. The autogenous mastoid cortical bone has been described as a satisfying and low-cost graft for minor ossicular defects [6,7]. On the other hand, sculpturing autogenous bone may be cumbersome and requires additional surgical time. Cadaveric homograft implants had ben employed until 1987, before being decommissioned due to the potential risk of infection [5]. In recent years, alloplastic prostheses have been designed with synthetic materials, such as metals, ceramics, plastics, and composites [1,8]. These prostheses are classified as Partial Ossicular Replacement Prostheses (PORP) when mounted upon the intact stapes, and Total Ossicular Replacement Prostheses (TORP) for cases with no stapes superstructure [9,10]. Currently, the most satisfactory alloplastic material in terms of hearing, lightness, rigidity, biocompatibility results, and easy surgical manipulation is titanium, used in ossicular reconstruction since the early 1990s [4,11,12]. Moreover, several authors have demonstrated the accuracy of MRI for residual and recurrent cholesteatoma to be higher in the presence of titanium than autologous bone prostheses [13]. However, titanium prostheses also present downsides, due to early and late postoperative extrusion. A recent meta-analysis reports extrusion rates of 9% for the PORP cohorts and 15.65% for the TORP cohorts [1]. Recently, new potential tissue bioengineering options have been explored, to aid in overcoming extrusion risks [14,15]. An interesting solution could be to combine conventional titanium prostheses with a coating of biological material, such as extracellular bone matrix. However, in the current literature, no bioengineering publications exist that explore the feasibility of such biohybrid prostheses. In this *in vitro* study, we seeded human adipose-derived mesenchymal stem cells (hASCs) onto titanium prostheses to verify that they support the colonization, proliferation, and osteoblastic differentiation of the cells, and we also verified the presence of bone matrix deposition. We aim to propose ossicular transplantation in order to obtain biohybrid prostheses, made up of titanium surrounded by bone extracellular matrix as bio-coating; here we present our preliminary results.

## 2. Materials and Methods

### 2.1. Production of Titanium Biohybrid Middle Ear Prostheses

**Figure 1:**
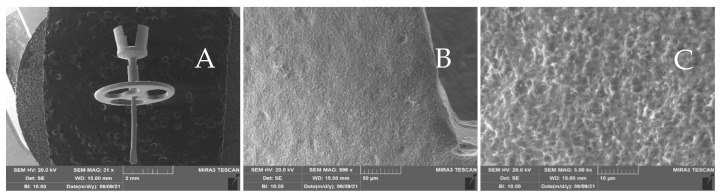
Scanning electron microscopy (SEM) study of the utilized prosthesis. Panel (**A**) indicates the structure and shape of the prosthesis (mag. 31×). Panels (**B**,**C**) show a higher magnification of the surface roughness (mag. 900× and 5.00 k×, respectively).

Scaffolds. The scaffolds used in this *in vitro* study were the titanium prostheses commonly used in our otosurgical department, and were gifted by Gyrus ACMI, Inc., Southborough, MA 01772, USA). The construct (Micron TM All Titanium PORP^®^ and TORP^®^, Gyrus ACMI, Inc., Southborough, MA, USA) was made of titanium (Ti6A4V ELI), with a size range of 2–5 mm (Figure 1).

Isolation of human adipose-derived stem cells (hASCs). Subcutaneous adipose tissue was obtained from the peri-trochanteric region of healthy donors during hip replacement surgery. Informed consent was obtained from all patients before surgery. The study was conducted according to the 1975 Declaration of Helsinki, and approved by the Ethics Committee of the San Matteo Foundation, Research and Care Institute, Pavia, Italy (P-20190023312, 9 April 2019). The samples were processed in the laboratory, following a precise protocol and maintaining sterile conditions. Briefly, the tissue was thinly chopped and subsequently incubated for 1 h at 37 °C in a shaking water bath with a digestion buffer (0.01% collagenase type II in DMEM F12-HAM medium). Afterwards, the collagenase was neutralized, and the suspension was filtered (using a filter of 100 mm) and centrifuged at 1200 rpm for 10 min at 4 °C. After two washes with phosphate-buffered saline (PBS) the pellet containing the hASCs was treated with a lysis solution, then suspended in a growth medium (GM, DMEM F12-HAM supplemented with 100 U/mL penicillin, 0.25 µg/mL amphotericin, 100 μg/mL streptomycin, and 10% FBS (Fetal Bovine Serum). The hASCs seeded in 75 cm^2^ polystyrene flasks were cultured in GM up to 95% confluence in a humidified atmosphere of 95% air with 5% CO_2_ at 37 °C. Thereafter, the adherent cells were trypsinized with Trypsin Ethylene Diamine Tetra Acetic Acid (EDTA), and 5000 hASCs/cm^2^ tissue culture plates were seeded in a new flask [16]. After repeating this step three times, a flow cytometer analysis was performed to observe the markers expressed by the hASCs. The analysis was negative for the hematopoietic cell markers CD34 and CD45, and positive for the mesenchymal stem cell markers CD73, CD90, and CD105 (Navios Beckman Coulter, Indianapolis, IN, USA). We used the Kaluza 1.2 software package (Beckman Coulter) to acquire, display, and elaborate data. The positive cells were counted and their flowcytometric signal was compared with the signal of corresponding immunoglobulin isotypes [17].

Cell seeding and culture on the scaffold. Given the shape and size of the construct (Figure 1), we chose the suspension seeding method to improve the cellular adhesion. Ten scaffolds were placed in a conical tube containing 250 μL of cell suspension (corresponding to 30,000 cells) and incubated for 24h at 37 °C under agitation in a humidified atmosphere of 95% air with 5% CO_2_, in order to allow the cells’ adhesion on the substrates. The next day the scaffolds were transferred into a 24-well plate and cultured in GM. After a week, half of the scaffolds remained in GM. In contrast, the other half were induced to an osteogenic phenotype by adding an osteogenic differentiation medium (OM, StemPro^TM^ Osteogenesis differentiation kit, Thermo Fisher Scientific, Waltham, MA, USA). After 14 and 21 days of culture, all cells seeded on scaffold constructs were sacrificed to carry on the experiments shown below. At the end of cell colonization, we would have a biocompatible structure composed of titanium coated with bone matrix secreted by cells previously seeded on the constructs.

### 2.2. Titanium Biohybrid Middle Ear Prostheses Analyses

#### 2.2.1. Cell Proliferation and Osteogenic Differentiation

Adhesion and Proliferation Assay (WST). To monitor cell proliferation, we analyzed the optical density at 1, 7, 14, and 21 days after the seeding of the hASCs on the ten scaffolds by using the WST method. This method uses cellular mitochondrial dehydrogenases to cleave the tetrazolium salt WST-1 in formazan. To evaluate cell proliferation, the Quick Cell Proliferation Colorimetric Assay Kit (Abcam, Waltham, MA, USA, #K301) was used, following the manufacturer’s instructions. The number of living cells is directly proportional to the amount of dye generated by the activity of dehydrogenase. The formazan dye produced by viable cells can be quantified by measuring the absorbance of the dye solution at 450 nm. The scaffolds were moved to new wells before the WST assay, to exclude the signal from cells that adhered to the plastic plates. After 7 days, the osteogenic differentiation of 5 scaffolds was induced, while the other 5 were kept in growth medium. The hASCs cultured on scaffolds, both in OM and GM, were incubated with 10% WST working solution at 37 °C for 2 h (95% air with 5% CO_2_), and then the absorbance was read using a spectrophotometer. 

RNA isolation and reverse transcriptase quantitative real-time PCR (qRT-PCR). To monitor the osteogenic differentiation, the gene expression of some known indicators of the osteoblast phenotype was tested. Then, 14 days after the use of osteogenic differentiation medium, RNA was extracted from the constructs with the Gene MATRIX KIT for RNA purification (Biosigma, Cona, Italy) to evaluate gene expression. The total RNA extracted was reverse-transcribed into cDNA using random hexamers and M-MLV Reverse Transcriptase. Quantitative real-time PCR (qRT-PCR) was performed in triplicate using 2 µL cDNA, obtained as above, using specific primers from Qiagen (Qiagen, Hilden, Germany) (Table 1): ALP (Alkaline phosphatase gene), RUNX-2 (Runt-related transcription factor 2 gene), COL1A1 (Collagen alpha-1-chain gene), OSX (Transcription factor Sp7), and BGLAP (Osteocalcin gene). The Quantifast-SYBR Green PCR Kit (Qiagen, Hilden, Germany) was used according to the manufacturer’s instructions, and qPCR was performed using the StepOnePlus™ Real-Time PCR System (Applied Biosystems™, Thermo Fisher Scientific, Waltham, MA, USA). The cycling conditions that we used to perform the qRT-PCR were the following: denaturation at 95 °C for 5 min; denaturation at 95 °C for 30 s for 40 cycles; 60 °C for 30 s for the annealing; and 72 °C for 40 s for the elongation. After the PCR run, in order to identify the melting temperatures of specific products, the melting curves were generated. The housekeeping gene β2M expression (beta-2 micro-globulin gene, Qiagen, Hilden, Germany) was used to normalize the qPCR reactions. The gene expression results of differentiated cells on the scaffold were expressed as fold change versus expression of hASC on the scaffold after 14 days of culture in the growth medium (control).

#### 2.2.2. Production of Extracellular Matrix

Immunofluorescence assay. The presence of collagen on the scaffold denotes the production of an extracellular matrix. It was demonstrated with an immunofluorescence assay to mark both phalloidin and COL1A1. Two scaffolds were evaluated, one cultured in GM and the other in OM. After 21 days from seeding, the cells grown on the scaffolds cultured in GM and OM were fixed with paraformaldehyde at 4% (PFA 4%) in PBS for 30 min. Cells were permeabilized with TRITON-X 0.4%, washed three times with PBS 1X, and incubated overnight at 4 °C with the diluted (1:50) primary antibody against COL1A1 (PA1-26204, Thermo Fisher Scientific, Waltham, MA, USA). Thereafter, the scaffolds were washed three times with PBS 1X and incubated with a secondary antibody—diluted 1:1000 (A-21207, Thermo Fisher Scientific, Waltham, MA, USA) for 30 min at RT. Then, the scaffolds were washed three times with PBS 1X and incubated with PHALLOIDIN (PHALLOIDIN Atto 488 Sigma, Aizu, Japan), following the datasheet. Finally, the scaffolds were mounted with an anti-fading mounting solution (ProLong^TM^ Gold Antifade Mountant Thermo Fisher Scientific) and kept at 4 °C until their visualization with the fluorescence microscope, Nikon Eclipse 80i (Nikon, Tokyo, Japan). 

Scanning electron microscope (SEM) analysis. The presence of the extracellular matrix on the constructs was evaluated through SEM on four scaffolds. After 21 days from differentiation, the constructs were washed with PBS and then fixed for 2 h with glutaraldehyde 2.5% in 0.4 M of Sodium Cacodylate Buffer. The scaffolds were subsquently washed with Sodium Cacodylate buffer for 30 min and then dehydrated with graded ethanol series, progressively increasing ethanol concentration from 50%, to 70%, to 90%, to 100%. A high-resolution SEM (EVO 40 SMART scanning electron microscope, Zeiss and TESCAN Mira 3 XMU, Pavia, Italy) was employed at 20 kV to perform the microstructural characterization. Two different SE (Scattered Electron) and BSE (Back-Scattered Electron) analyses were performed. SE uses secondary electrons emitted by the affected material to obtain a high-resolution three-dimensional image, while BSE uses the interaction between the electrons and atomic nuclei of the analyzed sample to create a grayscale image. Before observing the scaffold with the microscope it was necessary to coat the constructs with carbon, using a Cressington carbon coater 208c (Cressington Scientific Instruments, Watford, UK). SEM analyses were conducted at Arvedi Laboratory, CISRiC (Centro Interdipartimentale di Studi e Ricerche per la Conservazione del Patrimonio Culturale), University of Pavia, Pavia, Italy.

## 3. Results

### 3.1. Cell Proliferation and Osteogenic Differentiation

**Figure 2:**
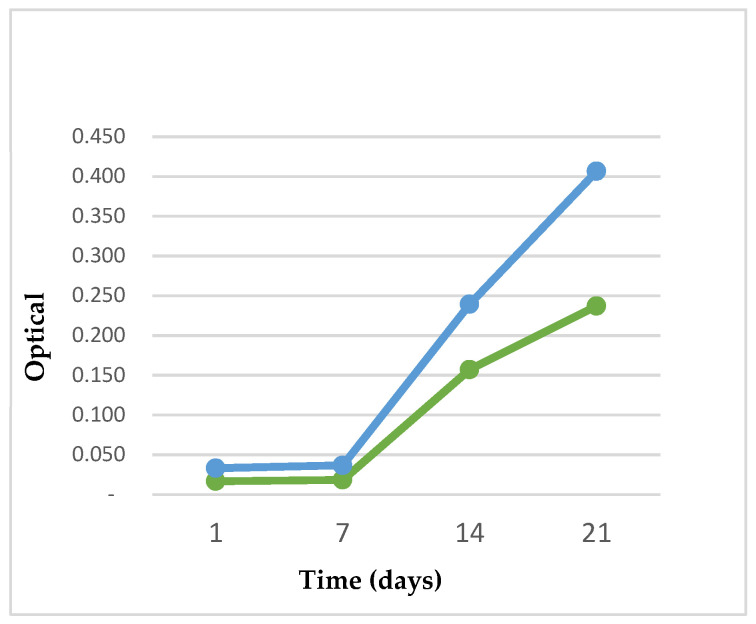
The graphic represents the values of the optical density at 1, 7, 14, and 21 days after seeding in growth medium (GM)—represented in the graph with a blue line—and osteogenic differentiation medium (OM), represented with a green line. The cell differentiation on each scaffold starts from 7 days after seeding.

Adhesion and proliferation assay. The cells do not show significative growth during the first week, but the numbers of cells are constant, probably due to their need to adapt to the new surface. From 14 days it is possible to observe a remarkable increase in the optical density of the scaffold cultured in GM, while a lower increase in optical density for the scaffold cultured in OM is in line with the concept that cell differentiation inhibits cell proliferation (Figure 2). 

**Figure 3:**
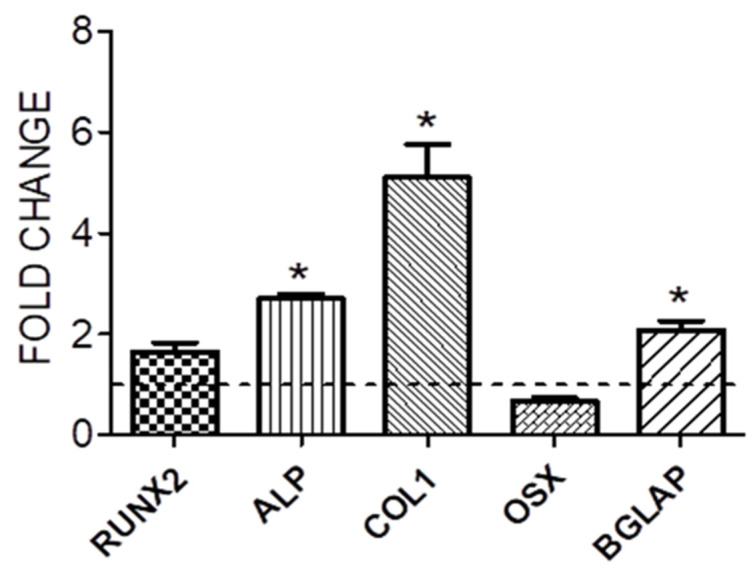
Gene expression of the hASCs seeded on the scaffold and cultured in the presence of osteogenic medium for 14 days. The gene expression results were expressed as fold change versus the expression of hASC seeded on the scaffold and cultured in the absence of an osteogenic medium (control). The dashed line represents the control. Bars represent the mean ± S.E.M. of at least 2 different experiments, each from different RNA extracts. * *p* ≤ 0.05 versus control. *ALP*, alkaline phosphatase; *OSX*, osterix; *BGLAP*, osteocalcin.

mRNA Expression. qRT-PCR analysis was conducted on four scaffolds with hASCs. According to the melt curve plot, there was only one peak corresponding to a single amplicon, indicating the specificity of the PCR reaction. The qRT-PCR results of the *Alp*, *Runx2*, *Col1a1*, *Osx*, and *Bglap* genes were expressed as fold change versus the gene expression of hASC seeded on scaffold and cultured in the absence of osteogenic medium (control). After 14 days, there was a significant increase in the gene expression of *Bglap*, *Col1a1*, and *Alp* (Figure 3) in constructs cultured in OM compared with control constructs.

### 3.2. Production of Extracellular Matrix

**Figure 4:**
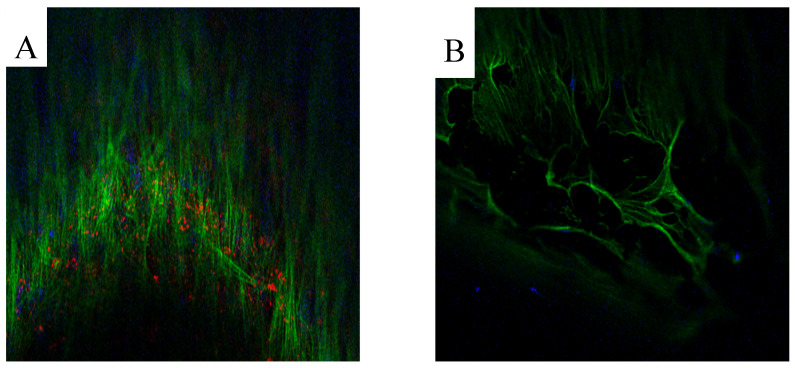
Immunofluorescence assay: (**A**) IF results of COL1A1 and PHALLOIDIN, conducted on a scaffold colonized with hASC differentiated in osteoblasts, mag 20×; (**B**) IF results of COL1A1 and PHALLOIDIN, conducted on a scaffold colonized with hASC cultured in GM, mag 20×. In the image the cell nucleus is marked in blue; the cytoskeleton (phalloidin) is marked in green; the collagen (COL1A1) is marked in red.

Immunofluorescence assay. The presence of collagen in the extracellular matrix secreted on two scaffolds—one cultured in GM and the other in OM—was evaluated through immunofluorescence assay. The deposition of collagen on the scaffolds cultured in OM was evidenced by the red staining, while it was absent on the scaffolds cultured in GM (Figure 4A,B, respectively). These results showed that the cells were able to differentiate in an osteogenic direction, and that they were able to deposit bone matrix on the structure’s surface.

**Figure 5:**
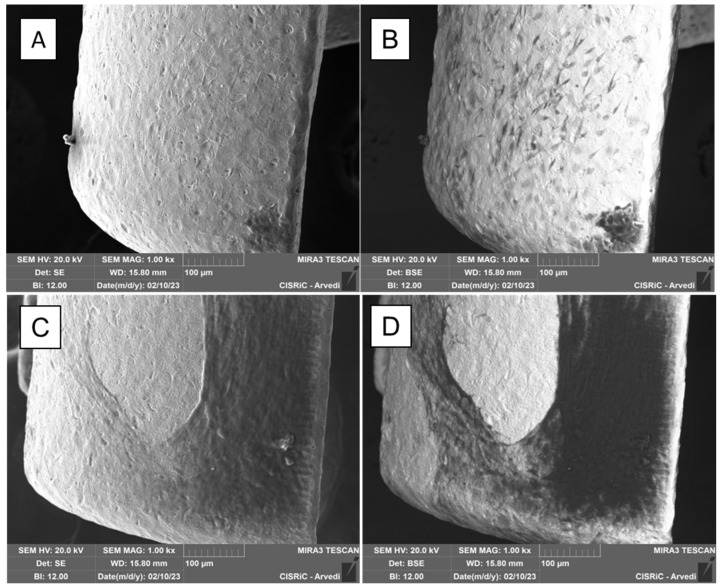
SEM images—taken with the scattered electron detector (SE) and the backscattered electron detector (BSE)—of the scaffolds cultured in GM (**A**,**B**) and in OM (**C**,**D**). With the BSE detector, cells and matrix appeared dark, prosthesis white. It is evident that scaffolds cultured in the presence of osteogenic medium are richer in matrix (**C**,**D**). Mag.: 1 k×.

SEM analysis. SEM analysis showed the presence and the colonization of many cells on two scaffolds grown in GM and two grown in OM medium (Figure 5). The BSE (on the right) allowed us to visualize cells and their matrix. The matrix and the cells appeared dark (organic zone), while the metallic scaffold appeared white (inorganic zone) (Figure 5B,D).

**Figure 6:**
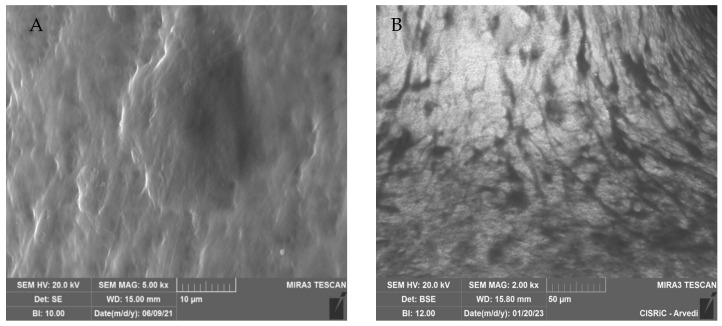
SEM analysis of matrix on the scaffold. The abundant matrix secreted by hASC differentiated in osteoblasts on the scaffold when observed with SE detector and Mag.: 5.00 k× (**A**) and BSE detector and Mag.: 2.00 k× (**B**).

The abundant matrix secreted by the hASCs, cultured in the presence of osteogenic factors and differentiated in osteoblasts on the prosthesis used in this article, was more visible at higher magnification both with the SE detector (Figure 6A, mag. 5000×) and the BSE detector (Figure 6B, mag. 2000×).

## 4. Discussion

ME surgery outcomes are undeniably influenced by the pre-existing conditions of the tympanic cavity and the clinical behavior of ME diseases. This tight clinical–surgical correlation has stimulated the development of many strategies for disease eradication and hearing rehabilitation over the years, including approaches that bypass ME sound conduction [18]. On the other hand, among the surgical solutions intended to restore a mechano-acoustic coupling between the tympanic membrane and the stapes footplate, the synthetic ME prostheses still play a prominent role in modern ME otosurgery, since they mimic the natural mechanics of sound wave transmission at this level [19]. Titanium is reported to be an excellent material for synthetic ME prostheses because of its intrinsic properties: it is light, rigid, non-ferromagnetic, easy to handle during surgery, and it is a good sound wave conductor. In addition to this, its employment has been recorded in both adult and child cohorts [1]. However, this material is encumbered with a significant drawback shared by all synthetic ME prostheses currently in use: it can be rejected by the host organism, and this phenomenon is known as extrusion [1]. Specifically, host tissues interact with the surface of the synthetic implant, inducing a foreign body response and inflammation [20]. Foreign body inflammatory response and consequent prosthesis extrusion have an important negative impact on the long-term outcomes of the OPL [14]. On the contrary, it has been observed that extrusion does not occur when biological materials are used [21]. For this reason, many authors advocate the possible inflammatory reaction, caused by the direct contact between the titanium prosthesis and the tympanic membrane, claiming cartilage interposition should be protective [22]. Unfortunately, the data regarding the beneficial role of cartilage protection are often conflicting [23]. In this respect, we can suppose possible confounding factors, such as ME pathologies and surgical techniques, should be considered. In recent years, tissue engineering studies focusing on improving bio-integration and preserving acoustic performance have started to appear in the literature [14,15,24]. To the best of our knowledge, there is no record of an ossicular replacement biohybrid prosthesis made up of titanium enveloped in bone matrix and living cells. Our experimental *in vitro* results represent the first feasibility study on this topic. The final aim is to obtain a biohybrid device able to lower the risk of extrusion and to maintain the intrinsic acoustic properties of conventional titanium prostheses. 

Our data showed that hASCs can adhere to and proliferate on the scaffold surface represented by titanium prostheses in use in our otosurgical department. Cells began to proliferate after one week of seeding, following a period of cell adaptation to the titanium surface. It is possible that an increase in the number of cells is not initially detectable because an equilibrium is reached between cell proliferation and apoptosis. The proliferation rate of hASCs on scaffolds cultured in an osteogenic medium was slower compared to those cultured in growth medium: this is because cells tend to inhibit proliferation in favor of differentiation. Gene expression was analyzed after 14 days of the differentiation induction both in GM and in OM. In particular, we investigated the expression levels of the following genes, which are indicative of bone differentiation: Alp, Runx2, Col1a1, Osx, and Bglap. 

Alp (Alkaline Phosphatase) has two principal roles during osteogenic differentiation: it is an important player during the first phase of differentiation and in bone mineralization, so it is considered both an early and late marker of differentiation. Runx2 (Runt-related transcription factor 2) is an early marker of bone differentiation, since it is the first transcription factor required for the determination of the osteoblast lineage, and its expression levels decrease in the mature osteoblasts: this is the reason why there are no differences between the cells cultured in GM and those cultured in OM [25]. Osx (Transcription factor Sp7) regulates the osteoblast differentiation, and it inhibits osteoblasts’ mature markers like Col1a1 and Bglap. Col1a1 (Collagen alpha-1-chain) is associated with osteogenic differentiation and mineralization, while Bglap (Osteocalcin) is involved in bone remodeling by using calcium binding, metabolism, and energy metabolism on osteoblasts [26,27]. The gene expression data show that the cells are in the intermedial phase of bone differentiation: there is a significant increase of Alp, Col1a1, and Bglap, but an unmodified expression of Runx2. In agreement with the literature, Osx expression does not show any modification compared to the controls, because it is a late marker of osteogenic differentiation [28]. We performed an immunofluorescence assay to mark both phalloidin and Col1a1. Phalloidin stains a polymerized form of actin (F-actin), the main component of the cytoskeleton, while Col1a1 is the main component of Collagen Type I, the most abundant form of collagen in the human body. Collagen is the main protein of connective tissues, including bone tissue. Phalloidin staining shows that the cells were viable at the time of fixation, while the presence of Col1A1 in OM and its absence in GM indicates the beginning of differentiation and deposition of the bone matrix in OM. Moreover, an abundant matrix was evident on the scaffolds cultured in osteogenic medium at SEM observation. 

The preliminary findings derived from our work seem to be encouraging; indeed, our data show good results regarding the adhesion, proliferation, and differentiation of hASC on titanium middle ear prostheses. However, corroboration from a bigger sample size and comparison with other titanium prothesis models are required to validate our results. In our opinion, the evidence supported by this study should be implemented with additional physical analyses, including evaluation of surface features. Prostheses’ bio-integration has been demonstrated to be strongly dependent on environmental protein absorption [29]. Hence, future research has to be focused on the characterization of interaction between constructs’ surface and proteins. In addition to this, functional investigations on the sound transmission properties of biohybrid products should be performed. Finally, we advocate further efforts to improve biohybrid prostheses manufacturing. An example could be the use of three-dimensional (3D) printing technology, which has been widely documented in the otoneurosurgery field [30]. We can postulate that the production of ossicular replacement biohybrid prostheses could be optimized by introducing devices made up of titanium, surrounded by a 3D-printed, self-absorbable superstructure facilitating the scaffold coating by hASCs, as a sort of bone transplantation. Studies *in vivo*, after *in vitro* implementations, are mandatory to test the constructs’ stability outside laboratory conditions and their resistance to surgical manipulation. Finally, we can speculate that the possible future clinical application would be a patient-specific treatment consisting of a three-stage intervention. The first step would be devoted to the hASCs harvesting using minimally invasive techniques, the second to the cell seeding and coating of the prosthesis by the ECM, and the final step to implantation [31].

## 5. Conclusions

To conclude, our results indicate that hASCs can adhere to and proliferate on the titanium scaffold surface that we used in this study. Moreover, the proliferation rate of hASCs on scaffolds cultured in OM was slower compared to those cultured in growth medium: this is probably due to the cells’ tendency to inhibit proliferation in favor of differentiation. 

The gene expression analyzed after 14 days revealed that the cells have begun bone differentiation, as suggested by the significant increase of Alp, Col1a1, and Bglap, although there was an unmodified expression of Runx2. The immunofluorescence assay and the SEM analysis showed the capacity of hASCs seeded on our scaffolds to secrete and deposit an abundant bone matrix. 

To the best of our knowledge, this is the first feasibility study exploring a biohybrid prosthesis composed of titanium surrounded by a bone matrix as a possible tissue engineering option for fabricating ME ossicle replacements. 

Our experience seems promising; further *in vitro* studies will be necessary to increase the data obtained, and animal studies will be required to evaluate the percentage of device expulsion and to test their *in vivo* application. Furthermore, clinical trials should be run in an attempt to establish in clinical practice these biohybrid protheses combining the advantages of both titanium and biologic tissues.

## Figures and Tables

**Table 1:** Primers used in real-time PCR experiments.

Gene	Target Transcript	Quantitect Primer Assay (Quiagen)	Amplicon Length
ALP	NM_000478	QT00012957	110 bp
RUNX-2	NM_001015051	QT00020517	102 bp
COL1A1	NM_000088	QT00037793	127 bp
OSX	NM_152860	QT00213514	120 pb
BGLAP	NM_199173	QT00232771	90 pb
β2M	NM_004048	QT00088935	98 pb

## Data Availability

Data are contained within the article.
